# St. Columba's Hospital, Home of Peace

**Published:** 1915-03-13

**Authors:** Herbert R. Arbuthnot

**Affiliations:** President. Swiss Cottage, N.W. (formerly Friedenheim Hospital).; Chairman. Swiss Cottage, N.W. (formerly Friedenheim Hospital).


### THE EDITOR'S LETTER-BOX.

### St. Columba's Hospital, Home of Peace.

*To the Editor of* The Hospital.

Sir,—We shall esteem it a favour if you will kindly permit us to make it known that the title of this hospital is now as above.

Our many friends and supporters will, no doubt, understand the reasons which prompted the Council in their decision to take this step.—Yours faithfully,

Aberdeen, President.
Herbert R. Arbuthnot, Chairman.

Swiss Cottage, N.W. (formerly Friedenheim Hospital).

